# Unveiling the impact: understanding long-term care workers’ experiences and their perceptions of resident challenges amidst the COVID-19 pandemic

**DOI:** 10.1186/s12877-024-05656-0

**Published:** 2025-02-17

**Authors:** Donna M. Halperin, Krista Whitfield, Julie A. Bettinger, Marian Orhierhor, Katherine L. Salter, Bailey M. Selig, Anna Mack, Brian R. Condran, Alexa Davis, Camryn Salyzyn, Janet A. Parsons, Melissa Kervin, Scott A. Halperin

**Affiliations:** 1 Canadian Center for Vaccinology, Dalhousie University, IWK Health, Nova Scotia Health, Halifax, NS Canada; 2 Rankin School of Nursing, St. Francis Xavier University, Antigonish, NS Canada; 3 Vaccine Evaluation Center, BC Children’s Hospital Research Institute, Vancouver, BC Canada; 4 Department of Pediatrics, University of British Columbia, Vancouver, BC Canada; 5 Departments of Pediatrics and Microbiology & Immunology, Dalhousie University, Halifax, NS Canada; 6 Nova Scotia Health Learning Institute for Healthcare Providers, Nova Scotia Health, Halifax, NS Canada; 7 Li Ka Shing Knowledge Institute, St. Michael’s Hospital, Unity Health Toronto, Toronto, ON Canada; 8 Department of Occupational Science and Occupational Therapy, University of Toronto, Toronto, ON Canada

**Keywords:** Long-term care (LTC) facilities, COVID-19 pandemic, Healthcare workers, Residents, Public health guidelines, Safety measures

## Abstract

**Background:**

During the COVID-19 pandemic, long-term care (LTC) facilities in Canada were confronted with many rapidly changing public health safety guidelines. Based on the guidelines, LTC facilities had to implement a series of virus containment and mitigation measures, presenting significant challenges for both workers and residents. This research aims to provide insights that could be used to guide improvements in the experiences of LTC workers, and of residents, in future pandemic crises.

**Methods:**

A qualitative multi-case study was used to explore the pandemic experiences of a demographically diverse group of LTC workers in Canada, focusing on how public health safety guidelines impacted them, and their perceptions of challenges faced by residents. Fourteen workers were engaged from facilities in Nova Scotia and British Columbia, which are regions distinct geographically and with differences in safety guidelines and implementation. Semi-structured interviews were conducted between April to October 2021. Using thematic analysis, we identified patterns within and across the interview transcripts.

**Results:**

The thematic analysis provided an understanding of the experiences and perspectives of LTC workers. There were four key themes: (1) *Tangling with Uncertainty*, that describes the effects of ambiguous messaging and shifting COVID-19 safety guidance on workers; (2) *Finding Voice*, that highlights how workers coped with feelings of helplessness during the healthcare crisis; (3) *Ripple Effects*, of pandemic pressures on workers beyond resident care, that included strengthening of inter-colleague support as well as financial challenges, and; (4) *Loss of Home*, where workers perceived that protection of residents led to a loss of the residents’ home environment, personal freedom, and autonomy.

**Conclusions:**

The findings suggest that LTC workers’ experiences during future pandemics may be improved by their inclusion in the development of public health safety guidelines, facilitating inter-colleague support systems, and ensuring worker financial stability. A balance should be found between preventing infection in LTC facilities and retaining the principles of holistic and resident-centered care for workers’ and residents’ mental health benefits.

**Supplementary Information:**

The online version contains supplementary material available at 10.1186/s12877-024-05656-0.

## Background

Severe acute respiratory syndrome coronavirus 2, or SARS-CoV-2, the causative agent of the global COVID-19 pandemic, first emerged in late 2019 in Wuhan, China [1, 2]. Groups most vulnerable to severe and symptomatic COVID-19 infection include adults over 60 years of age [3, 4], especially those with underlying health comorbidities such as respiratory, cardiovascular, neurological and immune diseases, and malignancies [3–7]. Long-term care (LTC) facilities typically care for seniors with underlying health conditions, and thus the residents have a disproportionately higher risk of serious and life-threatening illness from this highly contagious virus [8] than the general population [3, 4, 9]. Before COVID-19, LTC facilities (or sometimes called LTC homes) were overcrowded with residents, underfunded [10] and experienced chronic shortages of staff and other medical resources [10–12], each of which was intensified during the outbreak [10, 11, 13]. It has been suggested that these pre-existing conditions within the Canadian LTC system (that in Canada includes residential care, continuing care and nursing homes [10, 14, 15]) may have contributed to the fact that more than 5,300 COVID-19 deaths occurred within LTC facilities during the first wave of the pandemic (by the end of May 2020 [11, 12, 16]). Further, Canada’s reported LTC resident death rate during the pandemic (by the end of 2021) was among the highest in the world, as evidenced by the fact that LTC residents accounted for only 3% of the country’s COVID-19 cases but comprised 43% of the deaths [10]. Together, the situation of high infection and mortality rates among residents in LTC placed extensive pressures on those working in LTC facilities, particularly given the already stressed conditions of the LTC system [10, 14].

The pandemic response in LTC within Canada included rapidly evolving, reaction-based (to the latest information on the virus’ frequency/transmission), and often mandated public health safety guidelines (PHSG), that varied by province/territory, whose governments regulate LTC systems and public health [10, 17] (note: herein, PHSG refers to governmental guidelines, while approaches at LTC facilities to comply with these guidelines are termed as measures, tactics and/or rules). PHSG were set by different regulatory agencies that had non-uniform structures, which depended on the province, and thus PHSG and LTC measures were non-homogeneous in timing and/or implementation [17]. In turn, LTC facilities worked to comply with the changing COVID-19 PHSG and implemented stringent virus containment and mitigation (safety) measures within their premises [10, 18, 19] such as obligatory masking and other personal protection equipment (PPE) rules, limitations on and cessation of visitation, mandatory COVID-testing, restrictions on staff contact with residents, the physical isolation of residents, and quarantines, creating an environment of continual change [10, 17, 18], and requiring ongoing adaptation by the workers. The rapidly evolving approach to the creation and revision of health guidelines and LTC measures runs counter to the notion that changes to safety guidelines should be communicated to health workers in a clear, timely, and transparent manner [20], leading to an atypical workplace environment for LTC staff, that presented unique challenges in their roles as residents’ caretakers [10, 19, 21]. While some studies have emerged on resident experiences in Canadian LTC during the pandemic (e.g [22–27]), much less is known about the impact of the pandemic, and particularly the strict and evolving PHSG and LTC measures, on LTC workers, including their perceptions on the effects on residents, with only sporadic research available to date (e.g [19, 21, 28]). Further research on this topic is warranted in order to provide a pathway towards improving LTC during future health emergencies. One suitable means to filling this knowledge gap involves the study of the lived experiences of those individuals who worked within LTC facilities and implemented safety measures during the COVID-19 pandemic, including consideration of how they responded and adapted to the evolving circumstances, and how the pandemic rules affected the lives of LTC residents from the workers’ vantage point.

The objective of the present investigation is to improve our understanding of LTC workers’ experiences, including their perceptions of resident challenges, during the COVID-19 pandemic and PHSG in Nova Scotia (NS) and British Columbia (BC), Canada. Using a qualitative multi-case study design [29, 30], we consider how the workers interpreted, implemented and worked with rapidly changing PHSG, explore which factors they suggest supported/impeded the implementation of specific LTC measures, and assess the workers’ perspectives on residents’ experiences. Workers’ experiences are characterized by four themes: (1) Tangling with Uncertainty, Ambiguous Messaging and Shifting Guidance; (2) Finding Voice: Coping with Helplessness in a Healthcare Crisis; (3) Ripple Effects: Pandemic Pressures Beyond Resident Care, and; (4) Loss of Home: The Delicate Balance of Protection and Freedom in LTC. Using insights provided from these themes, we discuss potential avenues to improve LTC during future pandemics.

## Methods

### Study approach

We used a qualitative descriptive study approach, which involves detailed descriptions of experiences and circumstances as perceived by individuals (an approach commonly used to study healthcare workers [30]), along with a multi-case design [29]. This methodology allowed us to examine how LTC workers interpreted, implemented, and were personally impacted by COVID-19 PHSG during the pandemic, as well as their perceptions of resident experiences. For the study, we targeted workers within the contexts of NS and BC, Canada. These two provinces are geographically separate within Canada and differed in the implementation and/or timing of the onset of COVID-19 PHSG and LTC measures [17, 31]. At the time of the study (in 2021), both provinces had rapidly ageing populations, with 21.8% and 19.5% of the populations of NS and BC being over the age of 65, respectively [32]. Each province has its own hierarchical public health structure, with a provincial public health officer, regional health authorities (BC) or zones (NS), local (municipal) leaders, and institutional leadership at LTC facilities, whereby COVID-19 PHSG were directed at the province-wide level affecting all LTC facilities [33, 34], and in turn, interpreted and put into action by LTC team members within each facility [18, 21]. Our study purposely encompassed variation in geographical location, cultural background, rural/urban settings, private/public LTC facility types, COVID-19 PHSG, the local decision makers that shaped the PHSG and LTC measures, and workers’ roles within facilities.

### Principles of the study design: defining the case

We primarily employed the multi-case study design according to Stake [35]. Stake [35] indicates that the case provides original insights into complex issues, including the researcher’s emic perspective, while also comprising an approach that is holistic, empirical, interpretive, and emphatic in design [35, 36]. Further, Stake’s [35] approach is inherently flexible and pragmatic [36], which allows for adaptations based on the research context, and we adapted the approach to focus on the case distinction, rather than interpretations. To support a flexible and adaptive approach to data collection and analysis in keeping with the rapidly evolving context of the COVID-19 pandemic, we incorporated the principles introduced by Merriam [37, 38]. A multi-case design was applied in order to facilitate comparisons between provinces [39] (NS and BC), given that they had different experiences in terms of COVID-19 infection, and in their PHSG and implementation of LTC measures [10, 17]. For our analyses, the overarching case context across both study sites was the global pandemic and its response, with each provincial region serving as a case. Cases were bounded by geographical region and the timeframe in relation to the evolving COVID-19 pandemic. Cases were compared by applying both within-site analysis to understand each case within their individual contexts and between-site analysis to identify similarities and differences between cases.

### Participant sampling and data collection

The LTC facilities in this study were identified from the authors’ discussions with colleagues who had professional contacts within the Canadian LTC sector and from publicly available information, such as LTC facility websites. Leadership within the selected facilities were contacted and supplied with electronic recruitment materials (via email), that were in turn distributed to facility workers. Participants sampled herein included healthcare aides, clinical nurses, licensed practical nurses (LPN), clinical leadership and management, support service providers and family physicians. For an LTC worker to be included as a participant in the study they had to meet the following criteria: (1) work at an LTC site under study; (2) be sixteen years of age or older; (3) speak one of the two Canadian official languages; and (4) freely agree to participate by giving verbal or written consent.

Individual, semi-structured interviews (guided and flexible) were conducted with fourteen participants. In NS and BC, interviews were conducted online using a secure end-to-end encrypted web-based platform (i.e., Zoom) during 2021. Interviews were conducted virtually, as in-person interviews and/or monitoring was not feasible under the COVID-19 pandemic measures, and because virtual interviews were in line with our goals of understanding participant experiences, and their perceptions of resident experiences under PHSG. Further, study of participants from virtual interviews during the pandemic allowed us to achieve real-time perspectives of participants, that is, reflections on their current or recent experiences. Four members of the study team conducted interviews across both study sites (KW, MO, MK; and JB (Acknowledgements)), with one to two team members chosen to interview each participant. Interviews were between 30 and 80 min in length. An interview guide (see Additional File 1) was developed to cover topics such as how the workers and the residents were affected by the safety measures; however, interviews were largely participant-led, and researchers sought to minimize their influence on participants’ responses. The interview guide was pilot tested with two NS work colleagues of the authors (one in Public Health; neither were included in the 14 participants), and adapted throughout the course of data collection to reflect the developing analysis [37, 38]. Notes and memos were taken by the interviewers during and after each interview. All interviews were audio recorded and transcribed verbatim by a professional transcriptionist. Team members reviewed each transcript for accuracy and de-identification. Participants were invited to review their transcript to ensure it was accurate and de-identified. Four participants reviewed the transcripts and made no changes.

### Data analysis

Interview transcripts were imported into NVivo (R1/2020) for management and coding. Thematic analysis of each transcript was conducted to identify, analyze, and report patterns within the data, as described by Braun and Clarke [39, 40]. This approach, when expanded to the study of all transcripts, promoted the discovery of how shared and individual experiences impacted the participant responses [41] to the strict COVID-19 PHSG and LTC compliance measures and shaped their services to the LTC residents. Analysis began with data familiarization that included reviewing notes, memos and transcripts, and an initial round of coding (BS, AD, CS, MO, JAB, AM, KW, BC). Once a preliminary codebook was established, multiple team members (BS, AM, CS, KW, BC, MO, JAB, KS, DH) contributed to an iterative process of analysis through subsequent rounds of collaborative coding and dialogue. The codebook was used to provide a dynamic and adaptable framework, evolving as the analysis progressed and as deeper insights were gained into the data. This adaptive approach aligns with Braun and Clarke’s [39, 40] emphasis on flexibility, by allowing codes to be refined or expanded based on the richness and complexity of the dataset. Further, the codebook ensured that all team members were aligned while allowing for the iterative and reflective process central to the aforementioned methodology [39, 40].

Individual researchers’ personal values and positionalities were carefully considered throughout the process to ensure that emerging themes accurately reflected participant experiences. This affirms reflexivity and transparency, which are key markers of quality in qualitative studies. Reflexivity, as defined by Tracey [42], includes reflection and awareness by researchers on their own values and potential biases, and the need for transparency in methods. Data from each site were initially analysed separately, followed by between-site analysis in order to identify shared themes, and to highlight the commonalities and differences among sites. Virtual meetings between researchers at the two sites functioned to triangulate investigator points of view, which solidify findings and strengthens credibility [42]. A thematic report was generated using the data from NVivo. The thematic report was refined through an iterative process, returning to the raw data when warranted, until a consensus was reached among the entire research team that all reported themes accurately represented the data from both study sites [42].

### Rigor and trustworthiness

We designed the study to align with well-established principles of qualitative research, using Stake’s case study methodology [29, 35] as the foundation while incorporating complementary approaches to ensure rigor and trustworthiness. To achieve this, we employed strategies such as triangulation, reflexivity, and member checking, as well as verbatim quotations that precisely reflect workers experiences/perceptions within each case, which provided a robust framework for interpreting the data within its unique context. These methods allowed us to maintain methodological integrity while capturing the richness and complexity of the case. For instance, within the multi-case design, we ensured there was wide diversity in the demographics, locations, and worker position types of participants sampled that functioned to establish rigor, robustness, richness and multivocality within our findings [42, 43]. In terms of data collection in interviews, team members that conducted the interviews had regular debriefings to discuss their thoughts and observations, as is recommended for qualitative rigor [42]. Detailed notes and memos were taken during and after interviews [44], an approach thought to provide reflexivity and to improve analytic insights of researchers [45]. Interview transcripts were re-evaluated in an iterative analysis to support thorough code and theme development, an approach conducive to enhancing rigor in multi-case studies and qualitative research [35, 46]. Further, we included verbatim quotations of participants in our study, that richly reflect an individual’s lived experiences using their own language, and adds to the rigor and understanding of a described event [47, 48].

In agreement with a rigorous multi-case study [29, 35], a direct line of evidence was established and preserved during all stages of analysis and interpretation [29, 49], including repeated comparisons to the study objectives. While we identified prominent or shared patterns within our data [29], there was also a careful consideration of divergent patterns, exceptions, alternate themes, and competing explanations [29, 50].

Together, our study approaches aimed to ensure methodological rigor and credibility in addressing our research questions: (1) What were the range of experiences of LTC workers during the pandemic, particularly in relation to PHSG and LTC safety measures? and (2) How did workers perceive the experiences of residents? By employing largely unstructured, participant-led interviews and implementing steps to recognize and limit researcher influence in processing transcripts and results, we aimed to rigorously capture the pandemic experiences in LTC from the workers’ perspectives.

### Author positionality

We engaged in reflexivity throughout all stages of the research. Individual researchers’ positionalities, including our professional and social identities, were carefully considered throughout the process to ensure that emerging themes accurately reflected participant experiences and to affirm sincerity of findings [42]. This was a collaborative team project. The study team included qualitative research staff (MO, BS, AM, BC, AD, CS, MK, JB), senior researchers (DH, JAB, KS, JP, SH), and a nursing student pursuing undergraduate studies at the time of data collection (KW). Several authors were experienced regulated healthcare professionals (DH, AM, AD, JP, SH), from the fields of nursing, rehabilitation and medicine. All authors involved in the interviews and in subsequent analysis had experience and/or training in qualitative research methods. In addition, all members of the study team except BC and SH were women and all had prior experience in policy-related health research. None of the authors had relationships with any of the participants prior to the study. Four members of the study team had prior experience working within LTC before (KW, BS, DH) and/or during (KW, AM) the pandemic. Each of the authors also had their own lived experiences with the pandemic as residents of Nova Scotia (KW, BS, AM, BC, AD, CS, MK, DH, KS, SH), British Columbia (JAB, MO, JB), and Ontario (JP) during the COVID-19 outbreak. Well-considered steps were taken by all the authors involved in analyses with respect to ensuring the findings and themes reflected workers’ perspectives, as described throughout our Methods, and particularly our “Rigor and trustworthiness” section. None of the authors, with the exception of JAB, were involved in the decision-making or implementation process of the PHSG during the COVID-19 pandemic.

## Results

The study sample of LTC workers consisted of fourteen participants: eight were from facilities in NS and six were from BC (Table 1). The interviews were held in NS from early September to early October 2021 (shortly after wave three of the pandemic [51]) and were conducted in BC from April to June 2021 (during the third wave [51]). The sample included participants from one of the following classifications of healthcare professions: direct patient care provider (healthcare aide, clinical nurse, LPN), clinical leadership and management, supportive services and a family physician and represented facilities that had, and had not, experienced a COVID-19 outbreak in their history (Table 1). The participants were diverse in terms of sex/gender, ethnicity, and age, but to maintain confidentiality we have not described these demographics in detail here due to the rural nature (and thus small populations) of some LTC facilities and local communities, where such information may identify participants. From our analyses, we identified four themes that described the experiences and perspectives of the participants (Table 2).

**Table 1:** Demographics of LTC workers interviewed for the present study. *N* = 8 participants in NS, *N* = 6 in BC

Characteristic				Classification of Worker				
		Direct Patient Care Provider		Clinical Leadership and Management		Supportive Services		Physician
**Province**								
Nova Scotia		5		2		1		0
British Columbia		3		1		1		1
**Urban/Rural**								
Urban		3		1		1		1
Rural		5		2		1		0
**Public/Private**								
Public		2		1		0		0
Private		5		2		2		0
Public + Private		0		0		0		1
Unknown		1		0		0		0
**Outbreak Status**								
Outbreak		2		1		1		0
Non-Outbreak		5		2		1		0
N/A		1		0		0		1

**Table 2:** The four themes identified from interviews with participant workers at LTC facilities

Themes Derived from Workers’ Lived Experience
Theme 1: Tangling with Uncertainty: Ambiguous Messaging and Shifting Guidance
Theme 2: Finding Voice: Coping with Helplessness in a Healthcare Crisis
Theme 3: Ripple Effects: Pandemic Pressures Beyond Resident Care
Theme 4: Loss of Home: The Delicate Balance of Protection and Freedom in LTC

### Theme 1 - Tangling with uncertainty: ambiguous messaging and shifting guidance

Theme 1 is summarized as tangling with uncertainty, that largely resulted from the ambiguous messaging and shifting guidance experienced by workers during the pandemic under the rapidly evolving COVID-19 PHSG and LTC measures [10, 11, 17]. These uncertainties arose from inconsistent messaging about the guidelines and about how to adapt the guidelines into LTC measures, as well as from the perceived compromises in residents’ care, as described in the following.

#### Uncertainty arising from frequent changes in safety guidelines and inconsistent messaging

Participants in our study described a heightened sense of uncertainty with respect to the rapidly-evolving COVID-19 PHSG, particularly about how they would be implemented in LTC (Theme 1, Table 2). For instance, some participants in NS and BC indicated that as the pandemic progressed, they relied on live news media outlets to receive guideline updates provided by Nova Scotia’s Chief Medical Officer and British Columbia’s Provincial Health Officer, respectively. However, each briefing seemed to provide different, and sometimes conflicting, sets of information. Participants experienced uncertainty about how best to stay updated on the latest COVID-19 PHSG and how to comply with those measures in LTC. A BC supportive services worker described this experience as “a bit of a guessing game” (BC128). Other participants from NS shared their challenges with frequent changes in policies.

*“And of course*,* you never knew day to day what was going to change. So that caused some stress and the fact that you didn’t know.” (NS39*,* Direct Patient Care Provider)*.

*“The policies changed on a daily basis in there. One day things would be one way. […] And what worked today got changed the next day. And they would*,* it was like everybody was running around like chickens with their head cut off there in the beginning.” (NS40*,* Direct Patient Care Provider)*.

The lack of consistency in the latest COVID-19 PHSG for LTC was exacerbated in BC, where participants indicated that different guidelines were communicated by various health authorities. For example, a participant from BC acknowledged their experience working under conflicting sets of policy information from the provincial and local governments.

*“The other interesting kind of thing I found here*,* working here*,* is that we hear from on TV about kind of the policies of [British Columbia’s Provincial Health Officer] saying’ care homes can do this and do that’*,* yet seemed like [our local health authority] was giving us different rules to follow.” (BC128*,* Supportive Services)*.

#### Uncertainty about how to adapt safety guidelines to fit in the context of LTC

The lack of clarity on the latest pandemic PHSG often left participants unsure about how to adapt and comply within the context of their LTC caretaker roles. Participants described feelings of uncertainty and expressed that there was a gap between the intent of the newest PHSG and their practical implementation within facilities. There was a need for adaptation, but they had insufficient guidance about how to comply with the guidelines.

*“So we were trying a little bit of information that we had. We had some direction from department of health. But you always had those questions*,* what if what if? […] So there was an awful lot of questions. And what’s the best practice here?” (NS41*,* Clinical Leadership and Management)*.

*“Um*,* also we get um*,* policies from—trickle from the Ministry of Health from the talks that they have twice—well*,* they had talks daily coming to us. And we would kind of get the idea*,* but we weren’t exactly sure how to implement them. Which could be quite confusing.” (BC132*,* Clinical Leadership and Management)*.

#### Uncertain and unrealistic: perceived compromises to resident care

Participants spoke about how the one-size-fits-all approach to PHSG (e.g., across hospitals and LTC) was unrealistic at the implementation level. Questions around implementation were characterized as not only increasing uncertainty but also as markedly increasing workloads. This prompted some participants to question the value of some of the PHSG. For example, although PPE precautions were intended to minimize the spread of COVID-19, to keep both workers and residents safe from infection, implementation of this safety measure did not take into consideration the repercussions on the residents and sometimes compromised their quality of care. For instance, the PPE requirements created delays, both in the fulfillment of daily care routines and in the staffs’ response to health incidents involving residents. A participant from NS spoke about these challenges.

*“I guess it became even more of a stressful and an irritation*,* I guess*,* because if you were with one client and you were working and then you heard somebody else shout*,* ‘I need a hand*,*’ you would have to de-robe*,* sanitize your hands*,* and all that and then get geared all up again for a five second tug up in bed. Whereas before you could just leave. Wash your hands*,* go in and do it. You had all these extra steps and Lord help us when somebody had a fall.” (NS39*,* Direct Patient Care Provider)*.

Further, the same participant noted that masking precautions appeared particularly unreasonable.

*“We were wearing masks and the residents weren’t*,* but they were also more vulnerable people. But it just seems some things are just ass backwards*,* you know*,* the cart before the horse.” (NS39*,* Direct Patient Care Provider)*.

Some participants from BC shared similar sentiments that reflected uncertainty around the rationale for the implementation, or de-implementation, of COVID-19 PHSG. In one BC site, participants felt the decision to reopen their LTC facility to visitors (that were largely tied to vaccination rates [52]) at the peak of their third wave of COVID-19 was surprising. There was an underlying uncertainty about the basis for this decision.

*“One of the things that was really hard for us was when we went through the third wave*,* when things—the numbers in the community really*,* really got high*,* the highest ever. That’s when we opened our doors up to visitors coming in the building. [laughs] That was*,* that was the absolute craziest one.” (BC132*,* Clinical Leadership and Management)*.

### Theme 2 - Finding voice: coping with helplessness in a healthcare crisis

The second theme is described as LTC workers finding their voices and coping with helplessness during the pandemic (Table 2). Helplessness, a feeling that has also been described as uselessness [53], has been conceptualized within the context of healthcare during the COVID-19 pandemic as negative psychological feelings related to powerlessness, where the latter results from one’s real or perceived lack of control over a situation or event, and an inability to enact change [53]. Helplessness was described among workers due to an array of pandemic-related circumstances within the LTC facilities.

#### Helplessness of workers was linked to LTC safety measures that restricted the provision of compassionate, resident-centered, care

Due to the COVID-19 safety measures at LTC facilities, participants described feelings of helplessness as they witnessed the suffering of residents, while being powerless to provide the same scope of care that they were accustomed to providing before the pandemic. Participants explained how the infection control measures placed limitations on the manner in which they could provide individual care during a time of substantial personal struggle among residents and that this was a difficult adjustment for them.

*“[…] just see them struggle through it*,* knowing that there was nothing that we could do*,* besides the basic needs. Was a big adjustment.” (NS39*,* Direct Patient Care Provider)*.

*“We still need to be able to offer the same care*,* but how do we do it?” (BC121*,* Direct Patient Care Provider)*.

Under the PHSG and LTC measures, the model of LTC care shifted from resident-centered (or person-centered) care focused on controlling the spread of COVID-19 infection. The prioritization of infection control and mitigation strategies often conflicted with the principles of resident-centred care typically used by LTC workers, whereby residents set their own schedules, decide day-to-day activities, and are involved in care decisions; factors that contribute to individual resident’s quality of life [54, 55]. Many participants highlighted how the medical-, or task-centered focus on infection control, prevented them from meaningfully integrating individualized care and compassion into practice.

*“[…] you’re not being hired to make a bed*,* you’re not being hired to give a medication*,* you’re not being hired to do an assessment. You’re hired to care for someone. […] They’re people. They’re not a task*,* they’re people.” (BC121*,* Direct Patient Care Provider)*.

*“So every opportunity*,* you know*,* it’s distance*,* distance when*,* you know*,* the role of a CCA [continuing care assistant] is care*,* compassion*,* love*,* support. So the people that you’re caring for and teamwork with the team that you’re working with.” (NS40*,* Direct Patient Care Provider)*.

#### Helplessness and the lack of personal and physical interaction with residents

Participants shared stories of connecting personally with residents prior to the pandemic. For example, they described sitting with residents to converse about their family, daily events, and to help identify their individual emotional or physical needs. During the pandemic, however, these types of conversations abruptly stopped under the imposed safety guidelines/measures due to lack of staff time available, potentially arising from an environment of limited staffing and due to the time involved in adhering to PPE rules. Participants described how they had limited time and opportunities to connect personally with the residents.

*“So back in the day*,* you know*,* you had time to sit down and chat with the residents about their family and what’s going on in their lives. Nowadays*,* nurses can only meet the physical needs of the resident…” (NS35*,* Supportive Services)*.

*“Cause if I am some of the last people they’re going to see*,* I don’t want them to just see me zip by them. ‘Hi*,* what’s up? But I don’t—I don’t have any time. Oh*,* no*,* I got to help this per—oh*,* this bell’s going*,*’ uh—no*,* no*,* no*,* no*,* no.” (BC121*,* Direct Patient Care Provider)*.

Many participants expressed their feelings of helplessness when witnessing the decline in health of residents. Participants did not have the time to attend to residents’ emotional or interpersonal needs. Under the COVID-19 PHSG and LTC measures, workers were unable to use therapeutic touch on patients, an approach normally employed to demonstrate compassion and empathy. Participants recounted the struggle of observing residents’ frustration and sadness while being unable to comfort and reassure them.

*“And if they were upset*,* needed a hug you give them*,* not so now unless you’re providing personal care. We weren’t to be touching. That*,* I think was the biggest hurdle for both staff and residents.” (NS39*,* Direct Patient Care Provider)*.

*“I miss being able to hug my—my—my folks that—that—I do [sic]… that human contact is something that we all miss and we’re—the whole world is missing that.” (BC121*,* Direct Patient Care Provider)*.

#### Helplessness, the lack of consultation on safety guidelines, and finding voice

Some participants described how the LTC workers had been largely omitted from helping in the decision- making during the development of COVID-19 PHSG and expressed an eagerness to contribute their first-hand knowledge in resident care to developing those guidelines that may be effective when applied in practice (that is, as measures in LTC facilities). In this sense, the participants expressed how they felt unheard and how their voices were not apt to lead to change, and they wished to help improve circumstances. They were largely impeded in their ability to help ensure that the COVID-19 safety guidelines were implementable and beneficial in practice.

*“[…] we should have been consulted on some of these directives before they were*,* the decisions were made. Right? Like some of these people in the Department of Health and Wellness have no long-term care experience. Yet they’re making decisions for us*,* you know*,* so we’re the subject matter experts. Use us. we’re willing. But we didn’t feel that.” (NS42*,* Clinical Leadership and Management)*.

*“So if they’re thinking that this needs to happen*,* then there should be some kind of dialogue*,* right. Um*,* you know*,* so people have a chance to say*,* look*,* no*,* actually if you did that*,* you would have this impact*,* right. […] And*,* you know*,* it just seems like there’s no uh*,* access to*,* you know*,* the*,* the people who sort of do the work day-to-day.” (BC130*,* Physician)*.

It is noteworthy that there were some cross case differences in LTC worker opportunities to express their concerns and provide feedback, and thus to find/release their voices. For instance, participants from NS indicated that they had few or no opportunities to express their opinions about the COVID-19 PHSG and LTC measures, while in BC, the industry funded non-profit association, SafeCare BC, was perceived as a safe forum for workers to share their experiences. A BC participant discussed SafeCare BC, and also a related online and phone forum called Care to Speak, where LTC workers were welcome to express their opinions and vent their concerns with others in the health community.

*“[…] Safecare is—is somebody that we work with on a constant basis. The health authorities did a great job with opening up*,* um*,* town halls*,* making that quite available. It’s—I believe they did three or four over the last year with care aides*,* nurses*,* uh*,* overall*,* all staff*,* just to give people an opportunity to—to have a forum to—to—to [sic] express their concerns*,* what’s going on. […] They have opened up a few forums so people can voice their concerns*,* they can vent. I believe that’s always a good thing.” (BC121*,* Direct Patient Care Provider)*.

*“But through our benefit package for counselling*,* through Safecare with their Care to Speak […] to give you an opportunity to voice*,* through the forums that are set up*,* through our*,* uh*,* site so that you can vent*,* uh*,* and share your opinions…” (BC121*,* Direct Patient Care Provider)*.

However, while these forums provided opportunities for participants to express their concerns and be heard and appeared to afford some sense of relief for BC workers, they did not provide a means to influence PHSG changes. Helplessness was a theme common in participants’ accounts across both provinces even though such forums were only available in BC (see examples above in this sub-section and other sub-sections in Theme 2).

### Theme 3 - Ripple effects: pandemic pressures beyond resident care

The impact of the pandemic on LTC workers was not limited to their workplaces but also had ripple effects that extended into their personal lives and their mental health and wellness (Theme 3). The participants expressed concerns regarding spreading the virus to residents, colleagues and family members, the importance of having supportive relationships with colleagues to cope with the stress of the job, and the financial burden of pandemic-related workplace regulations. These aspects are elaborated in more detail below.

#### Workers’ fears about transmitting the virus

Participants described persistent fears about COVID-19 transmission. Specifically, they were concerned about transmitting the virus from the facility to their home, and vice-versa, potentially risking the health of LTC residents, coworkers, family members, and loved ones.

*“We’re just making sure*,* doubly sure that we are keeping COVID out. And that was the biggest thing is we didn’t want to have what happened at [LTC Facility] happen at [LTC Facility]*,* because [it] was just devastating.” (NS38*,* Direct Patient Care Provider)*.

“*I think my staff was really affected. They were terrified of bringing it into the facility. They were also terrified of if it was in the facility to go and bring it back to their families*,* yeah yeah*. *Both ways*,* just terrified either way*,* you know*,* yeah. Just being the transmitter*,* you know.” (BC132*,* Clinical Leadership and Management)*.

The participants explained how they had to trust that all their co-workers were adhering to pandemic guidelines both within and outside of the LTC facility to prevent transmission. They emphasized how it would take only one person to start an outbreak, and how trust amongst their colleagues was instrumental in dealing with the stressful, unpredictable and long-term situation of the COVID-19 pandemic.

*“[…] we had to learn to trust our co-workers and say*,* OK*,* like you have to be careful*,* you have to practice social distancing. You have to practice good hand hygiene*,* stay within that family bubble in the house.” (NS36*,* Direct Patient Care Provider)*.

#### Pandemic-related pressures improved inter-colleague support and connectedness

Participants described how their pandemic-related stresses and experiences improved inter-colleague support and strengthened the connectedness among staff [19, 56]. The staff relied on each other when seeking virus safety measure information, personal support, and solace. Participants described how this informal inter-colleague support system helped provide a source of emotional strength, allowing space to share their fears and other emotions during the pandemic. Further, participants explained that coworkers became like family, and that their shared difficulties helped to unite them as a team, and this was observed in both provinces under study, and for different worker position types.

*“[…] we developed a stronger bond within the—during this COVID period. Because I don’t know what I know—where I work*,* we also had an outbreak*,* very bad one*,* so it kind of brought us together. […] So we—many people—the way you can only relieve your fears*,* anxiety is by talking.” (BC123*,* Direct Patient Care Provider)*.

*“I think the best thing that came out of this pandemic was coming together as a facility and getting to know my coworkers. I can compare it to almost going through a tragic event with somebody.” (NS35*,* Supportive Services)*.

Colleague support and connectedness was particularly beneficial when considering the participants had never experienced a pandemic at this scale and had many unanswered questions around the nature of the virus. Participants reflected on how they discussed these unknown circumstances with each other and referred to the collective “we” and “all of us” when discussing their fears.

*“[…] And it was the front lines. it was very scary. It was almost you know*,* I don’t want to compare it to a war zone because that would take away from what our veterans do. But it was*,* you know*,* almost equivalent because we felt* *like we were* *fighting this unknown*,* silent killer. And it was scary for sure.” (NS35*,* Supportive Services)*.

*“Yeah*,* and it’s become hard for all of us* *and again*,* goes back to a lot of the staff having to take stress leave just because it’s so much with home and work […] And we all say*, *like*,* when will it end?” (NS39*,* Direct Patient Care Provider)*.

Given the pressures on workers in their daily lives, the development of informal collegial support systems was a resource on which they relied for mutual support and comfort.

#### Personal financial security was affected by pandemic public health guidelines for some workers

To reduce the risk of transmission among the vulnerable LTC resident population, some provinces including BC and NS imposed a one-worksite order [17], that stipulated workers could only work at one LTC facility. Some participants spoke about how this new rule resulted in a reduction in work shifts and a loss of income, and led to worries about supporting themselves and their families.

*“I think on the staff level*,* some of them that worked fewer shifts*,* they had two [LTC} homes that they were working at and they had to choose which one they were going to work at because they couldn’t work at two. […] Financially*,* you know*,* and in some incidents*,* you could tell those that were really struggling and fed up […] it really hit the pocketbook and how secure you felt.” (NS39*,* Direct Patient Care Provider)*.

*“I had a casual job to supplement income*,* but when the outbreaks started in March last year*,* they had to stop us doing more than one job*,* so we all fell under the one worksite.” (BC123*,* Direct Patient Care Provider)*.

### Theme 4 - Loss of home: the delicate balance of protection and freedom in LTC

Many participants shared a belief that while LTC facilities are workplaces they also comprise a permanent home for the residents, and thus should reflect a home-like environment [57]. Theme 4 explores participants’ perspectives on how residents experienced restrictions to their personal freedoms, autonomy, and the loss of their “home” under the COVID-19 LTC safety measures. In addition, this theme considers the tensions participants faced when implementing rules intended to keep residents physically safe from COVID-19 infection while also aiming to retain the residents’ well-being and their sense of a home. A participant from NS spoke of these challenges, saying:

*“Rather than just looking at the physical needs of somebody. We have to look at their physical*,* social*,* emotional*,* spiritual and mental healthcare needs of these clients for sure.” (NS35*,* Supportive Services)*.

#### Lack of interpersonal connections and autonomy for LTC residents

Participants described the changes that were made in the LTC facilities to comply with COVID-19 PHSG and to minimize spread of the virus that directly affected the residents’ home environment, such as moving residents into different rooms to make space for COVID-19 isolation quarters and confining the residents to their rooms within the facilities. Many participants also spoke about how implementation of these LTC safety measures resulted in the loss of freedom for residents to roam and to maintain interpersonal connections among each other and with staff, as they had done before the pandemic. Moreover, the measures led to substantial limitations on the residents’ autonomy in terms of making decisions about who to spend time with, when to interact, and in what activity.

*“And then we had to have isolation rooms*,* so we had to move residents around*,* we had to move residents out of rooms*,* into different rooms and have isolation rooms*,* quarantine rooms on every unit […] When they are only allowed in there and they’re allowed out of there*,* nowhere else in the building. All of that.” (NS40*,* Direct Patient Care Provider)*.

*“So*,* like*,* um*,* I know people in single bedroom rooms*,* they just kept them in their rooms and didn’t let them out.” (BC132*,* Clinical Leadership and Management)*.

Participants also shared how group recreation programming was cancelled under LTC facility measures to comply with distancing and gathering COVID-19 PHSG. The cessation of recreational and celebratory activities resulted in the loss of a major source of group interactions among residents, and of their connections among familiar people. Further, in some facilities, residents could no longer visit other residents in separate segments of the building.

*“They always had the big monthly birthday bash and there was bingo on Thursdays and then they did like smaller portable*,* small teas where they would do teas*,* cookies or whatever*,* and someone would play a violin or guitar or whatever and they could sing songs. And there was the trivia group and those just all stopped*,* abruptly stopped. But then there was nothing to do. And you couldn’t go visit so-and-so down the hall because you had to stay on your own wing.” (NS39*,* Direct Patient Care Provider)*.

A participant from NS described the detrimental consequences of discontinuing recreation activities for residents with dementia, stating:

*“We include our residents with dementia in our programming. And sometimes it might be just coming down somebody who is fairly advanced in the disease and doesn’t independently interact. If we’re doing baking we will often bring them down and bring them in the room. And even the smells and the textures of what’s being made connects them*,* right. So you take away all these things and all these connections that that keep them connected to a time and place and person and self.” (NS40*,* Direct Patient Care Provider)*.

#### Visiting restrictions impaired family connections and isolated residents

During the pandemic, strict limitations were placed on residents’ guests who visited LTC facilities under the COVID-19 rules (for example, they could not leave and return to facilities with a resident, and could not roam the facilities) that further contributed to the isolation of residents [10]. These restrictions were later followed up with a ban placed on visits to LTC facilities [17]. Participants expressed that being able to welcome visitors, especially family members, is central to the concept of home for residents, and this was inhibited (and sometimes prevented) by the rules. The participants explained how during the initial phase of visitor limitations (before banning) they were placed in the challenging situation of having to explain the necessity of the visitor rules to frustrated residents and family members.

*“[…] you’d have somebody that had a visit with their family*,* but they weren’t able to go with their family*,* whereas before they might have been able to go out for coffee or go for supper or go for a drive*,* then we’re dealing with increased behaviors that get to a point where we need to possibly medicate. And how fair is that?” (NS38*,* Direct Patient Care Provider)*.

*“[…] on the kind of same time with the visitations*,* having to really explain to families*,* you know*,* when it started off*,* you were able to visit*,* to visit [sic] – able to visit in the room […] but you can’t wander around in the building*,* you can’t say hello to other residents or go sit in the dining room with them and stuff.” (BC128*,* Supportive Services)*.

Participants further recounted that during the subsequent banning of visitors, residents living with cognitive impairments experienced confusion, and detailed how they needed to reassure and help redirect those residents.

*“And to uplift*,* kind of keep their spirits up because like working with Alzheimer’s and dementia*,* they didn’t quite understand. And we’ve just*,* oh not to worry*,* honey. You’ll see your son or you’ll see your daughter. […] So that’s just to kind of keep their hope alive.” (NS36*,* Direct Patient Care Provider)*.

*“Some of them didn’t understand why their family couldn’t come […] Um*,* they didn’t know why they couldn’t go out um*,* just to go*,* go out to their family’s place.” (BC132*,* Clinical Leadership and Management)*.

#### PPE and quarantines isolated residents and contributed to the loss of a home environment

Participants described how the implementation of PPE protocols reinforced the sense of loss of home among residents. These pandemic measures were stark reminders to residents of the loss of pre-pandemic familiarity and comforts of their home, and not only created physical distance, but also acted to impede communication between residents and staff members. Some participants recalled incidents where residents struggled to recognize staff wearing masks, while others spoke about the dehumanizing effect PPE had on residents. Caregivers who once felt like family members to residents now appeared as indistinguishable strangers.

*“Like if you had a roommate*,* that was who you could talk to*,* my staff and the cleaning staff that was coming in and out*,* but they were all garbed up in full PPE and it was just off putting right. And as well*,* having to wear the mask for any of them that are hard of hearing. […] And it was very frustrating to them.” (NS39*,* Direct Patient Care Provider)*.

*“[…] all of us in a mask and gown. We all look the same at the end of the day.” (BC121*,* Direct Patient Care Provider)*.

Additionally, mandatory quarantines, that were intended to limit the spread of COVID-19 in facilities, subjected residents to extended periods of solitary confinement. One participant recounted the intense fear observed in a resident at the thought of being quarantined and likened the experience to living in a prison, reflecting a growing sense of institutionalization within the facilities.

*“[…] if they had to go to the hospital*,* they had to come back and do a 14-day quarantine in an isolated room before they can come back into their room. And for some they said that was worse than prison […] One guy didn’t want to go to the hospital after he had to do one of those quarantines ‘cause he says*,* “I don’t want to do that again. I’d rather die.” (BC132*,* Clinical Leadership and Management)*.

Taken together, under the pandemic rules, the LTC residents experienced a marked decline in their autonomy and personal freedoms. Residents were often confined to their rooms, isolated from the staff (intensified by PPE and limited resident-centered care), and prevented from having in-person interactions with family/loved ones and other residents, shifting the atmosphere of their home to one of a rules-based institution [58, 59]. To our participants, the implementation of some of the pandemic measures appeared to be isolating and detrimental to residents’ mental and physical health.

*“Uh*,* it’s*,* it’s so isolating socially um*,* to*,* to be on a lockdown in a facility. Uh*,* to be in your room all day*,* have no activities. Um*,* and just have masked and gowned people sort of looking after you all the time*,* right. And yeah*,* it was*,* it was tough I think for a year uh*,* for those people. And many has passed away from just*,* you know*,* the*,* the social aspect wasn’t there for them. And they just couldn’t handle it right.” (BC130*,* Physician)*.

*“So the visitor restrictions*,* I think*,* were the were the biggest hurdle to kind of live with and deal with and help our residents get through because they suffered*,* like you could see it every day and they weren’t*,* they may might not have been dying from COVID*,* but they were dying because of COVID.” (NS38*,* Direct Patient Care Provider)*.

Participants described how LTC safety measures in facilities were designed to minimize disease transmission, yet unfortunately, some of the rules had unintentional consequences on the residents’ quality of life, with some residents being isolated for up to a year at a time. While participants understood the reasons for such protocols, they questioned whether the benefits ultimately outweighed the consequences for residents.

*“Yeah*,* they might get physically sick. But does that outweigh*,* them connecting with their family and friends and are we willing to take that risk?” (NS35*,* Supportive Services)*.

*“I wish there had been better ways of doing things that—for the people’s qualities of—quality of life in the care homes […] some care homes*,* residents*,* you know*,* maybe they had to be isolated from their families for three or four months*,* whereas other—others*,* it was*,* like*,* you know*,* a year and a bit.” (BC128*,* Supportive Services)*.

#### Participants developed adaptive approaches to interact with residents

Participants recognized the loss of the residents’ prior degree of interpersonal and physical interactions, and changes in their home environment, under the LTC safety measures and felt that the rules prevented them from providing their normal level of comfort and compassion to those under their care (see also Theme 2). Under this atmosphere, workers developed new approaches to communicate with and help residents. As an example, despite mandatory masking concealing most of staffs’ faces, they learned to use exaggerated expression to connect with residents.

*“So all of a sudden I*,* and I found we all laughed we would make our eyes wrinkle on the sides so people knew we were smiling at them. Otherwise you’re just a set of eyes with a mask over it.” (NS41*,* Clinical Leadership and Management)*.

*“[…] residents had to learn to read us too ‘cause they couldn’t*,* they couldn’t see all our expressions. So we had to learn to*,* to express things rather than just have it on our faces.” (BC132*,* Clinical Leadership and Management)*.

Given that the COVID-19 LTC measures acted to limit resident interactions among each other, participants developed creative solutions to help regain some sense of normalcy – that is, as much as possible under the circumstances. Although recreational programming activities were limited due to gathering restrictions, participants explained how they adapted certain activities to offer some degree of social engagement.

*“[…] we had to adapt a lot of the programs to meet the needs while still following protocol. […] So instead of having 30 people at church*,* we would have church at three different locations with 10 people on each of their cohorting units.” (NS35*,* Supportive Services)*.

*“[…] our recreation department did their absolute best with trying to come up with different ways to keep people engaged and people*,* you know*,* they might not have been able to sit all at the same table together. But there was always arts and crafts. They were baking stuff. […]. So we tried to maintain some sort of normalcy for their benefit.” (NS38*,* Direct Patient Care Provider)*.

#### Participants devised innovative tactics for resident visitation

Another action that participants undertook within the overriding context residents’ loss of home, and during the phase when visitors were banned, was devising innovative tactics that allowed family and loved ones to visit residents, while still adhering to the LTC safety measures. For example, one approach was window visitations (involving face-to-face visits across a glass window). Window visitations became popular among residents and allowed them to experience personal connections. To facilitate this form of visiting, a participant from NS shared how one colleague made every effort to provide this option to residents. Window visits were also described for residents in BC.

*“[…] the director of food services*,* actually changed her office to another spot because the window she has in her office is from the front of the building. So that was like a place for the residents to go. We had a phone inside and then a phone outside and they talked to each other and they had like a window visit in the beginning because we wanted to get that started.” (NS42*,* Clinical Leadership and Management)*.

*“[…] we did have virtual visits too and window visits as much as we could um*,* with families and friends. Um*,* our telephone was used a lot. Um*,* our portable phone was used a lot just to connect people.” (BC132*,* Clinical Leadership and Management)*.

Unfortunately for staff and residents, in compliance with changing COVID-19 PHSG and tightening restrictions, window visitations were eventually discontinued in an aim to keep residents and the visitors safe. Participants reported that rules for window visits were not always being followed and some people were passing items through open windows, as described by a NS Clinical Leadership and Management worker: “So we were kind of defeating the purpose of being apart by opening some of those windows and passing stuff back and forth.” (NS41). Other LTC facilities discontinued this practice due to community spread of the virus and because window visits became very busy which was impacting the safety of residents as well as the visitors. In response to the banning of window visitations, the LTC staff then turned to technology to mitigate the isolation residents were experiencing from the loss of visitations. Participants shared how integrating video chatting into residents’ day-to-day routines provided an alternative to in-person visits and facilitated some degree of connection and normalcy for residents and their loved ones.

*“Um*,* other families were delighted that the people that lived a great distance away were able to do a Zoom meeting with*,* with their loved one or Facetime. And normally they wouldn’t be able to do that*,* that. So that was good for families like that. And some residents were just delighted going*,* ah*,* look*,* my son.” (BC132*,* Clinical Leadership and Management)*.

While many participants emphasized that virtual visits were useful for some residents, others described the use of technology as a challenge. For example, a number of participants explained that residents with cognitive impairments often struggled to understand the physical distance between themselves and visitors on the screen.

*“But then again*,* is the issue with people that have any kind of dementia*,* they’re not understanding that they can’t touch their loved one through the screen or they can’t hug them ….” (NS38*,* Direct Patient Care Provider)*.

“*With some people with dementia they didn’t quite connect with the person on the tablet. So we just used the phone ‘cause people understand the phone more*,* yeah.” (BC132*,* Clinical Leadership and Management)*.

Another concern that arose from participants regarding technology was that not all family and loved ones had the resources or knowledge needed to facilitate a virtual visit. Particularly, older adults who were unfamiliar with video chat platforms were not able to join residents, an obstacle not typically observed among younger visitors. The following was indicated by a participant from NS.

*“[…] because a lot of older generations*,* older family and friends don’t have iPads*,* don’t have computers. So they weren’t actually able to virtually connect with their loved ones.” (NS35*,* Supportive Services)*.

One participant from NS working in supportive services reported that their LTC facility received funding to aid with creative strategies devised to promote recreational activities. They explained how the funds were used to buy iPads and hire additional staff such as support aides and music therapists. Moreover, funds were used to design a program whereby residents enjoyed virtual reality goggles to explore their old familiar neighborhoods on Google Street View.

*“We had an innovation project that I headed up and we got about eighty thousand dollars for a two-year pilot project for music therapy and palliative care. And so we use that money to do programs such as our virtual reality program. […] we were able to fulfill some of the social aspect that we were missing from the COVID-19 pandemic” (NS35*,* Supportive Services)*.

The same participant also shared how this funding was obtained due to a dedicated administrator and director of nursing who prioritized the value of recreation therapy in the health and well-being of residents.

*“I worked at another long-term home for March*,* April and May of this year*,* And when I talked to that department their jaw dropped open when I said we had this many support aides and this is what we did over the pandemic. And the director of recreation said to me*,* where did you get the money for this? Where did you find the resources and how they didn’t have any idea that they were available to use.” (NS35*,* Supportive Services)*.

No other participants in our study mentioned seeking or receiving additional funding for their facility. This may indicate that some facilities were unaware of available funding opportunities. In this regard, there may have been some degree of inequity in technological solutions for residents among facilities.

Even though creative solutions to visits and recreation may have allowed participants to extend some degree of compassionate care, one participant from NS also noted that the effort added stress during a time when they wanted to prioritize LTC safety measures.

*“It made the staff very creative and we had to remind them*,* you know*,* you are doing an amazing job. But it added another stress to being creative on top of a time when people just wanted to focus on being safe.” (NS41*,* Clinical Leadership and Management)*.

Taken together, the findings indicate the loss of a home environment by the residents was widely recognized by workers, and that the workers made substantial efforts to provide resourceful solutions to return a sense of normalcy to those under their care.

## Discussion

In our exploration of LTC workers’ lived experiences at facilities in NS and BC, Canada during the COVID-19 pandemic, we describe the impact of PHSG and implementation of measures in LTC on workers’ practices and well-being, and their perceptions of the impacts on residents. LTC workers consistently described the need for continuous adaptation to evolving guidelines that was linked to pervasive feelings of uncertainty (Theme 1) and helplessness (Theme 2), the latter reflecting workers’ lack of engagement in helping shape PHSG, and the limitations imposed on resident care under the LTC measures. In addition, many participants expressed how the pandemic affected their daily lives, including fears about transmitting the virus and financial instability under some safety measures, and described the strengthening of inter-colleague support and connections that helped them to cope with their ongoing stresses (Theme 3). In terms of LTC residents, participants recounted that from their viewpoint, the residents experienced an abrupt change in lifestyle, leaving them largely isolated, without resident-centered care, and lacking autonomy (Theme 4), which was an unintended consequence of pandemic measures. Some workers responded by developing innovative approaches to improve the residents’ home environments. Reflecting on and understanding the experiences of workers during the pandemic, and particularly under the evolving COVID-19 PHSG and stringent LTC measures, provides a meaningful opportunity to improve worker and resident outcomes in LTC during future pandemic crises.

### Heightened uncertainty and helplessness among workers during the pandemic

LTC staff across both study sites described their constant struggle interpreting and implementing the frequent changes in COVID-19 PHSG. The ongoing changes were largely due to the rapidly evolving information on the virus, such as its transmission pathways and/or positive cases in the population [17] and the increasing vaccination rate in the population, the latter which allowed for some stringent safety measures to be relaxed at the later pandemic stages. In addition, some workers described the arbitrariness of the timing of certain PHSG, such as re-opening of facilities to visitors during COVID-19 wave, causing confusion and uncertainty about PHSG decision making. In fact, the timing of visitor bans (and release of bans) varied across Canadian provinces [17], even though COVID-19 was spreading across the country in roughly similar time frames (albeit, with delays in surges in some provinces versus others, and staggered introduction of cases in some regions due to public health measures or the isolation of regions/communities), and PHSG appeared overall not to be timed with respect to pragmatic responses.

In our study, the rapid evolution of safety guidelines in BC and NS left LTC staff searching in real-time for up-to-date information, from various sources including public news conferences by Health Officials and from other LTC staff, which contributed to their uncertainty regarding both interpretation and implementation of safety measures in practice. Uncertainty in healthcare workers arising from complex and unpredictable situations, including COVID-19, has been linked to psychological responses such as fear and anxiety [19, 60–62], consistent with sentiments provided by LTC staff who participated in our study, which they described as stressful. Experiences of stress may have been due to pre-pandemic LTC conditions such as insufficient staff training in infection control, understaffing, overcrowding of residents, and limited PPE, all of which intensified during the pandemic [10, 11]. Overall, the reactive and evolving nature of the COVID-19 PHSG and LTC compliance measures, as well as the high contagiousness [8] and unknown/unpredictable nature of the SARS-CoV-2 virus itself, appears to have contributed towards the ongoing uncertainty and fears experienced by LTC workers.

For many participants, the lack of access to inclusive, bidirectional communication, through which consultation in guideline creation and/or provision of feedback could occur, contributed to a sense of helplessness. Helplessness (as previously indicated, has sometimes been described as uselessness [53]), may include feelings of being unable to do any more for a patient/resident, and has been linked to feelings of powerlessness to influence and authorize changes in a situation, such as COVID-19 PHSG and LTC measures [53]. Lack of feedback about health guidelines (to decision makers) from front-line health workers may hinder patient care [63, 64], as was suggested here by workers’ frustrations around the increased response times to patient needs created by PPE rules and by the prevention of personal interactions with residents under COVID-19 distancing measures. In hindsight, workers suggested that some pandemic guidelines/measures were unscientific, infeasible, and/or unnecessary at the LTC facility level. Workers in BC valued the opportunity to express their opinions on their experiences and to “vent” though SafeCare BC (and the related Care to Speak; similar outlets were not described for NS), which appeared to provide them with some sense of being heard, but these outlets were not designed to lead to changes in guidelines. Helplessness was a theme common to both BC and NS with respect to the lack of worker involvement in shaping safety guidelines (Theme 2), suggesting the presence/absence of these discussion outlets may not have influenced workers overall experiences around helplessness. In fact, overall, our results showed high similarity between the experiences of LTC workers between BC and NS (outside of the availability of forums for workers to vent or provide feedback, described only in BC), that points to a common problem, that being, the uncertainty around PHSG and their implementation in both regions of Canada. Together, these patterns suggest future response to pandemics, and particularly for highly contagious diseases such as COVID-19 [8], should include more avenues for LTC workers to provide meaningful input and feedback to policy decision makers in ways that will help to facilitate the creation of guidelines that reflect practice contexts.

As revealed through our participants’ accounts, safety measures markedly and quickly shifted the priority of LTC away from resident-centered care towards a medical and task-centered model, changing from holistic care to a biomedical care philosophy. Resident-centered care aims to promote each residents’ autonomy, choices, and personal strengths, with an overall goal of improving their quality of life and health [54, 55, 65]. Unfortunately, rules on distancing and PPE made integrating this resident-centered philosophy into daily care infeasible for LTC staff, contradicting their training, as they could not comfort, interact with, and assist the residents under their care in the ways they had prior to the pandemic. Together, a valuable lesson of the COVID-19 pandemic response is the core values of LTC care, particularly resident-centered care, need to be more thoroughly considered and made a stronger priority within future pandemic PHSG, to ensure the well-being of both LTC workers and residents.

### Personal effects of the pandemic response on workers

Participants recounted how their daily lives were affected during the pandemic. Working in a high-risk environment like LTC means there is an ongoing concern about spreading disease, given the often-unavoidable close contact required to provide care to residents. Further, this concern of workers may be exacerbated by the medical vulnerability of LTC residents, and the potential for the virus to lead to their hospitalization or death [11, 66]. The fear of disease exposure and transmission has been linked to increased levels of stress and emotional exhaustion (burnout) among healthcare professionals [67]. Importantly however, there may be avenues for remedy: for instance, it has been found that the occupational stress and negative psychological impact on front-line staff involved in infection control may be reduced when workers have been provided adequate training/preparedness and have prior experience in managing similar crises within healthcare organizations [68]. Thus, work organization is highly relevant to pandemic response, and it has been inferred that those workers with prior outbreak-experience should be strategically placed within the front lines and to handle direct patient contact during future disease outbreaks [68], that may provide a meaningful means to improve LTC workers’ experiences in forthcoming pandemics.

Another personal impact included workers’ concerns about financial instability resulting from pandemic protocols, and an inability to provide for themselves and their families, indicating this was a source of stress that extended into their personal lives. One contributing factor to the financial concerns mentioned in our interviews was the provincial one-worksite order and the associated limitations on the number of shifts available to workers, a concern that has been previously noted for workers in BC [28]. It appears reasonable to propose such financial impacts to workers may be avoided by aiming to provide income guarantees for staff, if/when such rules may be applied in similar future crises. This approach is similar to the strategy of providing workers with financial incentives/security in order to retain competent health staff when there are shortages of workers within the health sector [69].

The emergence of strengthened inter-colleague support and connectedness among LTC front-line staff was noted by workers studied here to provide a significant avenue of relief and understanding, a factor that may have helped to mitigate personal stress and fears (see also similar patterns in workers’ strengthened connections [19]). Reliance on coworkers appears to have been an outlet to relieve daily frustrations caused by the unpredictability of the day-to-day work environment, and the challenges of being impeded in applying their full training as caretakers. Such ethical dilemmas in health workers (wanting to help, but prevented from helping and/or using their full training) has been described as moral adversity, and may lead to moral distress and to mental disorders in workers (e.g., anxiety disorder, depression [70]). It has been suggested that during times of stress, social capital, defined as resources embedded within social structures to be used for action, has the potential to enable resilience among healthcare professionals; this worker connectedness may also facilitate valuable information sharing and support [71], the extent of which may depend on the organizational structure of workers and the degree of communication within and among branches of the hierarchy. Despite the sometimes-overwhelming challenges of LTC staff during the pandemic, their resilience, defined as the capacity to cope and manage under stressful conditions [19, 72], was observed from their determination to provide the highest quality of care possible while still abiding by safety measures. Further, resilience arising from inter-colleague support may help alleviate the moral adversity and distress of health workers (and thus mental health disorders), including during COVID-19 [70]. As has been suggested by Hung et al. [19], further research will be needed to ascertain precisely how LTC decision makers can encourage team resilience via structural supports in the workplace. The present study suggests inter-colleague support systems and resilience emerged naturally and informally among workers within LTC facilities, without structurally-based instigation. In this context, one potential straightforward approach to promote inter-colleague support and connectedness, as well as shared resilience, may be through LTC leadership providing time and physical spaces for staff to interact and discuss their shared experiences during future pandemics, as well as mental health and wellness services and counselling support systems (cf [73]). To prepare for future pandemics, such plans for LTC staff support should be set in place well in advance of the next crisis [10]. Our results suggest that a valuable area for future research should involve revealing how resilience emerged naturally and informally in LTC facilities, and whether this was dependent on workers’ physical proximity (to their colleagues) during their shifts, and/or included workers at different hierarchical levels in the organization.

### Loss of home for LTC residents

From the viewpoint of participants, the measures taken at LTC facilities to adhere to COVID-19 PHSG had negative effects on the well-being of LTC residents, described here as the loss of home (Table 2). Within an LTC facility, a sense of home for residents may be promoted by forming genuine relationships with staff, maintaining strong communication with family and friends, enjoying the companionship of other residents, and the presence of communal areas, all of which are thought to improve resident connectedness to their place of living [74, 75]. The limitations on movement within the facilities and on family visitations during the pandemic were especially devastating to LTC residents [76]. Family members may act as caregivers and advocates [10, 24], and thus their absence may exacerbate social isolation, loneliness, and accelerate mental decline in those with cognitive conditions [18, 77, 78]. It is worth mentioning public health guidelines initially put limitations on visitor numbers or movements within facilities, which was later followed up by the banning of visitors [17], suggesting the guidelines were initially formed with some attention to the importance of visitors to LTC residents’ well-being (by not banning visitors immediately). Our interviewees consistently recognized the effects of the loss of visitation on residents. Participants also indicated mandatory face masks and other PPE diminished residents’ ability to communicate, to understand and bond with staff, factors that may have further impaired the residents’ sense of familiarity and quality of life [77]. Taken together, participants suggested the pandemic measures left residents isolated and lonely for months at a time, a circumstance that should be improved and allayed in future pandemic emergencies. This can be achieved by learning from the voices of LTC workers about implementation of COVID-19 PHSG, and investing in pre-planning of pandemic guidelines that retain some degree of normalcy for residents.

Further to the isolation of LTC residents, workers described that the residents had minimal opportunities to receive compassion from staff, which is a fundamental aspect of holistic care, and contributed towards their loss of home. Compassion encompasses values of sympathy, empathy, and respect and the will to provide relief against suffering [79]. Providing compassionate care by workers involves having a conscious awareness of these values, and integrating them into practice by taking verbal, nonverbal and/or physical action to alleviate suffering [80]. The use of touch within healthcare practices includes both instrumental touch during patient care and expressive touch, the latter being intended to communicate compassion [81]. From our study, participants described that distancing precautions and PPE protocols prevented these practices, and they felt it added to residents’ isolation and loneliness, a situation that as above-mentioned, may give rise to moral distress of LTC staff [70]. It may be surmised that the lack of routes for staff to demonstrate compassion during the pandemic, and the loss of those personal interactions, contributed to the perceived reduction in the well-being of both residents and workers.

An important factor participants associated with the residents’ isolation was the marked decline of personal autonomy. Deciding day-to-day activities, such as when to invite visitors, interactions with family and friends, if or when to leave the room or facility, and to participate in decisions on one’s medical well-being, are all integral to a sense of autonomy and control over one’s life and feeling at home in one’s own space [75]. Personal autonomy, as aforementioned, is also a fundamental aspect of resident-centered care [54, 55, 65]. The LTC workers’ descriptions of a perceived decline in the mental and physical well-being of residents was intertwined with the nearly complete loss of their autonomy and freedom to interact with others. Some participants questioned whether the benefits of the LTC safety measures were outweighed by their devastating effects on residents’ lives. We suggest, as has been noted elsewhere, PHSG should aim to attain a better balance between protecting residents from infectious disease and the mental, physical, and social well-being of residents, including their autonomy, as a more humane and holistic approach in future pandemics [82].

Even before the COVID-19 pandemic, LTC facilities in Canada and elsewhere had been sometimes characterized as institutions, leaving staff struggling between the concept of “home” and “institution”, where the latter can be described as a facility where individuals are isolated and controlled by regulations/rules of an administrative system, rather than by their own personal choices [58, 59, 83]. As was described by the participants here, residents at LTC facilities were under the control of measures installed based on PHSG, such as mandatory quarantines, removal of social activities, forbidden visitations with family/friends, and restricted movements between different rooms and regions of facilities. These types of constraints on personal freedoms suggest the environment became more institutionalized under the implementation of safety measures [58, 83]. In this context, the retention of a sense of home, or normalcy, and avoiding the unintentional rule-based drift towards institutionalization, should be a greater priority in future pandemics. In particular, in hindsight, it may be suggested that there should be pre-pandemic planning and a focus on retaining only those rules with evidence of meaningfully benefiting workers and/or residents (that may differ from rules effective in other settings such as acute care and hospitals), particularly the stringent rules about visitors and staff PPE, that led to isolation of residents.

#### Innovations by workers to enhance residents’ well-being

It is worth noting that in response to the suffering and loneliness observed in residents, some LTC workers described how they and other staff developed innovative approaches to return a degree of positivity and normalcy, and a semblance of home, for the residents while adhering to pandemic measures. These types of novel methods have also been reported for front-line staff in other healthcare environments, such as in critical care in hospital settings, where staff caring for patients with cardiac conditions developed creative verbal and nonverbal solutions to interact with patients to meet their interpersonal needs [84]. For the LTC workers studied here, they created interpersonal communication approaches for residents such as video calls, window visitations, novel recreational activities, and exaggerated physical expressions when wearing PPE, all of which they believed helped improve resident morale. Technology based video visits with family/friends has been suggested to be an especially helpful source of comfort and normalcy and reducing isolation for LTC residents [78]. Our study suggests the use of technology for both visits and for recreation was highly beneficial to residents’ well-being, but these options were not available for all. For example, video calls are dependent on availability of funding for laptops, tablets or smart phones, that may preferentially exclude facilities in rural areas (that tend to have lower financial resources [85]), and relied on family/friends also having access to required technologies. Only one facility in our study had participants who described seeking and receiving additional government funding for technology-related recreational activities, suggesting an uneven knowledge of alternate funding sources or differences in capacity to pursue such options. It has been suggested one potential option to improve resident well-being during pandemic health emergencies in the future may be for governments to provide incentives and/or subsidies to technology companies that engage in increasing the capacity of LTC facilities to supply and use smart devices [78]. Approaches by government agencies may include offering the funds in a low barrier manner (including simple and low barrier funding applications), and widely publicizing funding availability for LTC facilities, and maintaining a baseline number of devices available in periods outside the pandemic within LTC facilities, so they are in place when one does arise.

#### Strengths and limitations

The present study has several notable strengths. First, we adhered to multiple well-established principles of qualitative research in our study methods and approaches to ensure rigor and trustworthiness of our findings and the identification of key themes (refs [43–48]). Second, we studied LTC workers and two different provinces in Canada, that differ geographically, have different health policy regulatory structures, and varied in the timing and implementation of PHSG [17, 33, 34]. This approach strengthens our findings that the pandemic response led to mostly shared common experiences among LTC workers, and how they perceived resident experiences, despite such differences in workers’ backgrounds (Table 2). Third, while conducting interviews during the pandemic was a challenge, given the stresses and the schedules of workers, the use of virtual interviews allowed a thorough and largely participant-led discussion that directly achieved our goals in revealing the effects of PHSG and LTC measures on their experiences, and their perceptions of resident experiences. Fourth, it was highly advantageous that our interviews were conducted during the pandemic, and thus the LTC workers experiences were current or recent to their recollections, and therefore could be recalled in (or near to) real-time, making them apt to be more precise than if interviewed post-pandemic; factors also adding strength to our conclusions (Table 2). In terms of limitations, we studied 14 individuals, that was a relatively modest sample size, and was potentially limited due the challenges in attaining volunteers during the pandemic, and this may have caused us to exclude some rare, but potentially meaningful, effects of the pandemic on LTC workers and/or their views on how it affected residents. In addition, the study of LTC facilities in even more provinces (than the two studied herein), may have helped reveal whether there were divergent experiences of LTC workers, and their perceptions of resident experiences, across other regions of Canada, and thus we stipulate that our present findings are limited to the provinces under study.

## Conclusions

This study provides insights into how LTC workers interpreted, implemented and worked under the rapidly evolving COVID-19 PHSG, including their perspectives on challenges faced by residents, and presented our findings into four themes. The themes included how workers tangled with uncertainty, found their voices under feelings of helplessness, the ripple effects of the pandemic on workers, that included strengthened colleague support as well as financial concerns, and the workers’ perceptions about the loss of a home for LTC residents (Table 2). The challenges faced by workers, and the ways they coped with and overcame those challenges, may help improve PHSG, LTC measures and workers experiences during future pandemic crises, particularly those involving a highly contagious agent such as COVID-19 [8]. While this study provides original insights into the direct links between specific types of pandemic policies (PHSG) and LTC measures (e.g., PPE, windows visits, banning visitors, lack of physical contact) on workers, and their perspectives of the effects on residents, other research has also recognized that COVID-19 policies were detrimental to the well-being and mental health of those living and/or working in LTC, and that improved policy responses are essential for the future (e.g [82, 86]). As an example, to enhance future responses, decision makers should consider establishing mechanisms for two-way communication to solicit input and actionable feedback by LTC workers on the creation and implementation of PHSG. In addition, improvements may involve the provision of physical spaces and time for the development of inter-colleague support systems and connectedness that facilitate well-being and information sharing, and the encouragement of worker-mediated recreational activities and resident visitations via technology. Many of our participants’ concerns revolved around observing the isolation of residents and the loss of resident-centered care and personal autonomy, that they felt led to declines in residents’ physical and mental health. In this context, rather than mainly focusing on physical health and preventing infection, as largely occurred under COVID-19 PHSG [10], future pandemic responses should prioritize a more balanced and holistic approach to resident care [82], that will benefit both workers and residents.

## Electronic supplementary material

Below is the link to the electronic supplementary material.

Supplementary Material 1

## Data Availability

As per our ethical approval and consent procedures, the transcripts generated and analyzed during the study contain sensitive, identifiable information. In order to protect the privacy of the participants, these transcripts are not publicly provided. However, we are committed to transparency and data availability in line with academic standards. The de-identified transcripts will be available upon reasonable request, subject to review and approval, in compliance with institutional ethics guidelines and privacy regulations. Requests can be directed to the corresponding author.

## Abbreviations

BC: British Columbia
CCA: Continuing care assistant
COVID-19: Coronavirus disease 2019
LPN: Licensed practical nurses
LTC: Long-term care
NS: Nova Scotia
PHSG: Public Health safety guidelines
PPE: Personal protective equipment
SARS-CoV-2: Severe acute respiratory syndrome coronavirus 2
