# Supplementary material for: Unveiling the impact: understanding long-term care workers’ experiences and their perceptions of resident challenges amidst the COVID-19 pandemic
Source: BMC Geriatr. 2025 Feb 17;25:108. doi: 10.1186/s12877-024-05656-0 (PMC11834567; doi:10.1186/s12877-024-05656-0)
Supplement: Supplementary file 1 — Supplementary Material 1 [file 12877_2024_5656_MOESM1_ESM.docx]

**Additional File 1** for

**Unveiling the Impact: Understanding long-term care workers’ experiences and their perceptions of resident challenges amidst the COVID-19 pandemic**

**Interview guide for LTC workers**

*[Consent and preamble to be read before starting the interview]*

| **TOPICS OF DISCUSSION** | **GUIDE QUESTIONS AND PROBES** |
| --- | --- |
| **Introduction** | As a start, tell us a little about yourself. Tell me about you, as much or as little as you feel comfortable.  *[Researcher Note: Leave it to the participants to present themselves in their own terms, if they struggle you may suggest talking about their professional role, who do they live with, what type or geographical area do they live in, what do they enjoy doing in their free time, or other.] Possible prompts below:* |
|  | **Queries**   - What is your role in [organization]? And how long have you been working in this role? - Has your role or responsibilities changed at all due to COVID-19? If so, in what ways? - What was your professional role? And/or what is your role now? How long were you working in that role? |
| **COVID-19 Policies implemented at the provincial and local level** | *[Researcher Note: The objective of this question is for participants to identify specific policies, and communications channels they use to learn about them. These examples will be used in subsequent questions]*  Since the start of the COVID-19 pandemic, there have been different policies implemented to help contain the spread of COVID-19 in Nova Scotia (*change for relevant locations*). |
|  | **Queries**  I am interested in what policies you noticed that have affected your workplace and/or clients in any way. Thinking back to the beginning of March (*change for relevant time at each location*) when policies first began to be implemented, can you tell me about the earliest policy or policies that stand out in your memory? As time went on, were there any other policies that stand out in your memory that impacted your workplace and/or clients?   - How were these policies communicated [within/to] your organization? - In your role, how do you usually seek out or receive information about policies that could impact your work? - What did you think about these communications? Among all the communications that were done during the pandemic, which ones do you remember that were either very clear or that left you confused? - What are your thoughts or perceptions about how these policies were communicated? |
| **Impact at the systemic level** | For the next series of questions, we will discuss the impact of the COVID-19 policies at various levels. To begin, we will focus on the systemic impact, which is the broader impact on institutions and groups. |
|  | **Queries**  From your perspective as a [role], what would you say were the biggest impacts of the policies you just mentioned?   - In what sense do you see that these policies have affected your province? - And in the locality you work, your city, what changes have you seen as a result of these policies? - More specifically, how have these policies impacted your work, I mean how your work was organized, and whether you were able to work? - How did the policies impact your ability/capacity to provide services to the communities you work with? - Did the policies change the services you provide? If so, how? |
| **Impact at the individual level (economic, health, social)** | Now, let’s talk about the impact of the COVID-19 policies at the individual level; specifically, the ways in which these policies have impacted you. |
|  | **Queries**  As [role] within [organization], how have these policies impacted your…   - Physical health, if you could give me a specific example that would be great. - Mental health, do you have a specific example or situation that illustrates this? - Day-to-day work, is there a specific example you could share? - Interactions with colleagues, if you could give me a personal example. - Personal life at home. - Are there other impacts you have experienced that you would like to share? |
| **Impact on groups experiencing vulnerability** | COVID-19 policies have impacted everyone, but not always in the same ways. Who do you think is being most negatively impacted by the COVID-19 policies? |
|  | **Queries**  From your perspective as a [role], who have you had contact with that you have seen, where they have been more negatively impacted than others?   - How were they impacted differently from other people you know? - What circumstances did they have that put them in a more vulnerable position? |
| **Agency: the ability to influence policy** | We have talked about the policies around COVID-19, and what impact you have seen at a broad level and at your more personal level. Now I’d like to ask you about whether you feel there were opportunities for you or others to influence these COVID-19 policies. |
|  | **Queries**  I am very interested in hearing your thoughts about how you, and your co-workers, have tried to *impact* policies (or would have liked to have influenced policy).   - Were there any opportunities to provide feedback on these policies before, during or after they were put in place? - Are there specific ways you’ve tried to adapt or influence these policies? - Is there a leader within your province, region, or a local organization, who you would have liked to sit down and discuss the COVID-19 policies with? For example, if you could have sat down with the Premier or the Chief Public Health Office of NS [*change for relevant locations*] to talk about COVID-19 (and the policies), what would you have wanted them to know? - How do you see the future? What would you like to see at the policy level? |
| **Wrapping up the interview** | Thank you so much for sharing with me your views and experiences. Is there anything else you would like to add? Is there any thought, or story that you would like to share?  We would like to send you a gift card as a token of appreciation. I can send it by email or by mail, are any of these options better for you? |

**Demographic Questions**

1. To which gender do you most identify?

- Male
- Female
- Transgender
- Agender
- Two-spirited
- Non-binary
- Prefer to self-describe: _____________________
- Prefer not to say

1. How old are you?

- Under 18
- 18-24 years of age
- 25-34 years of age
- 35-44 years of age
- 45-54 years of age
- 55-64 years of age
- 65-74 years of age
- 75 years of age or older
- Prefer not to say

1. What is the highest level of education you have achieved?

- Elementary school
- High school
- College
- University: 🞎 Certificate 🞎 Bachelor’s degree 🞎 Master’s degree 🞎 Doctorate
- Other, please specify: __________________________________________
- Prefer not to say

1. What was your employment status before the COVID-19 pandemic (prior to March 2020)?

- Working full-time
- Working part-time (less than 30 hours per week)
- Working casual
- Not currently working
- Student
- Retired
- Other, please specify: ___________________________________________
- I prefer not to answer

1. What is your current employment status?

- Working full-time
- Working part-time (less than 30 hours per week)
- Working casual
- Not currently working
- Student
- Retired
- Other, please specify: ___________________________________________
- I prefer not to answer

1. [Professional/Policymaker] What is your professional role? ________________________________
2. [Professional/Policymaker] How many years have you been in your current position? ___________
3. [Professional/Policymaker] How many years have you been in your profession? [If not relevant for participant, ask]: How many years have you been working in this organization?____________
4. What are the first three letters/numbers of your postal code? _______________________________
